# Cerebral microstructural alterations as an imaging biomarker for Post-COVID-condition

**DOI:** 10.1038/s41598-025-18962-3

**Published:** 2025-09-12

**Authors:** Alexander Rau, Philipp G. Arnold, Sibylle Frase, Nils Schröter, Hansjörg Mast, Cornelius Weiller, Marco Reisert, Horst Urbach, Jonas A. Hosp

**Affiliations:** 1 Department of Neuroradiology, Faculty of Medicine, Medical Center–University of Freiburg, University of Freiburg, Freiburg, Germany; 2 Department of Diagnostic and Interventional Radiology, Faculty of Medicine, Medical Center–University of Freiburg, University of Freiburg, Freiburg, Germany; 3 Department of Neurology and Clinical Neuroscience, Faculty of Medicine, Medical Center–University of Freiburg, University of Freiburg, Freiburg, Germany; 4 Department of Stereotactic and Functional Neurosurgery, Faculty of Medicine, Medical Center–University of Freiburg, University of Freiburg, Breisacher Str. 64, 79106 Freiburg, Germany

**Keywords:** Diffusion microstructure imaging, COVID-19, Post-COVID-condition, Microstructural MRI, Neuroimmunology, Neurological disorders

## Abstract

To develop an imaging biomarker-based approach for the diagnosis of Post-COVID-condition (PCC) at the individual patient level. Magnetic resonance imaging (MRI) data from a prospective cohort of PCC patients (n = 89) were compared with a control group of unimpaired individuals who had contracted coronavirus disease 2019 (COVID-19) in the past (n = 38). Participants were divided into two groups: a training and a test cohort. The macrostructure, diffusion tensor imaging, and multi-shell-based microstructure imaging metrics were extracted using an atlas-based approach. These data were subsequently utilized to train a linear support vector machine (SVM). The efficacy of discrimination between the groups was evaluated for various combinations of input parameters. Upon comparison of the different input combinations, we found the highest area under the receiver operating characteristic curve (AUROC) for microstructural parameters. For the optimal combination of input parameters, an AUROC value of 0.95 with a sensitivity of 94% and a specificity of 85% was achieved, indicating high discriminatory potential but also underscoring the need for further validation given the non-negligible false-positive rate. The atlas regions with the highest discriminatory power include both gray (including multiple cortical areas, putamen and left thalamus) and white matter (including corpus callosum and frontal white matter). The use of a SVM allowed for the differentiation between PCC patients and UPC participants with high sensitivity using microstructural MRI data. While these findings mark a significant step toward a biomarker-based diagnosis of PCC, the moderate specificity and the monocentric design emphasize the need for confirmation in larger and multicentric cohorts before clinical application.

**Supplementary Information:**

The online version contains supplementary material available at 10.1038/s41598-025-18962-3.

## Introduction

It is estimated that between 6 and 10% of patients who have contracted coronavirus disease 2019 (COVID-19) will experience a “Post-COVID-19 condition” (PCC)^1,2^. Fatigue and neurocognitive deficits are among the most frequently occurring symptoms associated with PCC and can have a significant impact on the disease burden^3^. The diagnosis of PCC is made in accordance with the criteria set forth by the World Health Organization (WHO)^4^: A probable or confirmed SARS-CoV-2 (Severe Acute Respiratory Syndrome Coronavirus Type 2) infection, manifested by at least one symptom with a relevant impact on everyday functioning, a persistence of the symptom for at least two months, and a delay of at least three months between the onset of acute SARS-CoV-2 infection and diagnosis. In the absence of an accepted pathophysiological disease hypothesis, PCC is a diagnosis of exclusion. However, this poses a considerable challenge in view of the substantial socio-economic implications, as the capacity to engage in gainful employment of those affected is impaired in approximately 6%^5^.

One method for objectifying the diagnosis of PCC would be to include imaging biomarkers. Based on magnetic resonance imaging (MRI), significant differences were identified on a group-level between patients with PCC and controls. Macrostructural alterations were observed, including a diminution in the volume of cortical regions, specifically the limbic system and the cerebellum^6^, as well as subcortical structures, namely the left thalamus^7^, putamen and pallidum. Diffusion tensor imaging (DTI) revealed not only reduced fiber integrity in white matter tracts, as evidenced by e.g. the corpus callosum and uncinate tract^6,8^, but also altered diffusivity in the left thalamus^7^. However, it is important to note that macrostructural and conventional DTI changes may reflect a general sequela of infection rather than a PCC-specific biomarker^9^. The use of state-of-the-art diffusion-based multi-shell protocols furthermore enables a comprehensive examination of the cerebral meso- and microstructure^10–12^. Thus, microstructural MRI parameters may provide more refined information on tissue composition and thus hold greater potential to discriminate PCC patients from unimpaired Post-COVID individuals. In line with this hypothesis, a recent study from our research group demonstrated pervasive alterations in cerebral microstructure, attributed to a shift in volume from neuronal compartments to free fluid, which were associated with the severity of the initial infection^13^. In terms of clinical outcomes, correlations were identified between altered imaging parameters and the symptoms of fatigue^7,13^, cognitive impairment^6,8,13^, and olfactory performance^13^.

Consequently, the objective of this study was to determine whether a pattern can be identified based on multimodal MRI data to enable an imaging-supported diagnosis of PCC at patient level, as to date most observations in PCC are made on group levels. For this purpose, we utilized a large prospective monocentric cohort of 89 patients diagnosed with PCC in accordance with the World Health Organization (WHO) criteria. The control group consisted of participants who have recovered from an initial infection with SARS-CoV-2 but were currently asymptomatic. A linear support vector machine (SVM) was trained to distinguish between the two groups. The input factors utilized in this analysis were as follows: 1 Tissue probability values (TPV) obtained by CAT12 to capture the macrostructure; 2 DTI-based indices; 3 Multi-shell derived parameters from neurite orientation dispersion and density imaging (NODDI), and diffusion microstructure imaging (DMI) to capture the meso- and microstructure. Also, we hypothesized that microstructural MRI parameters outperform macrostructure in diagnosing PCC.

## Methods

**Fig. 1:**
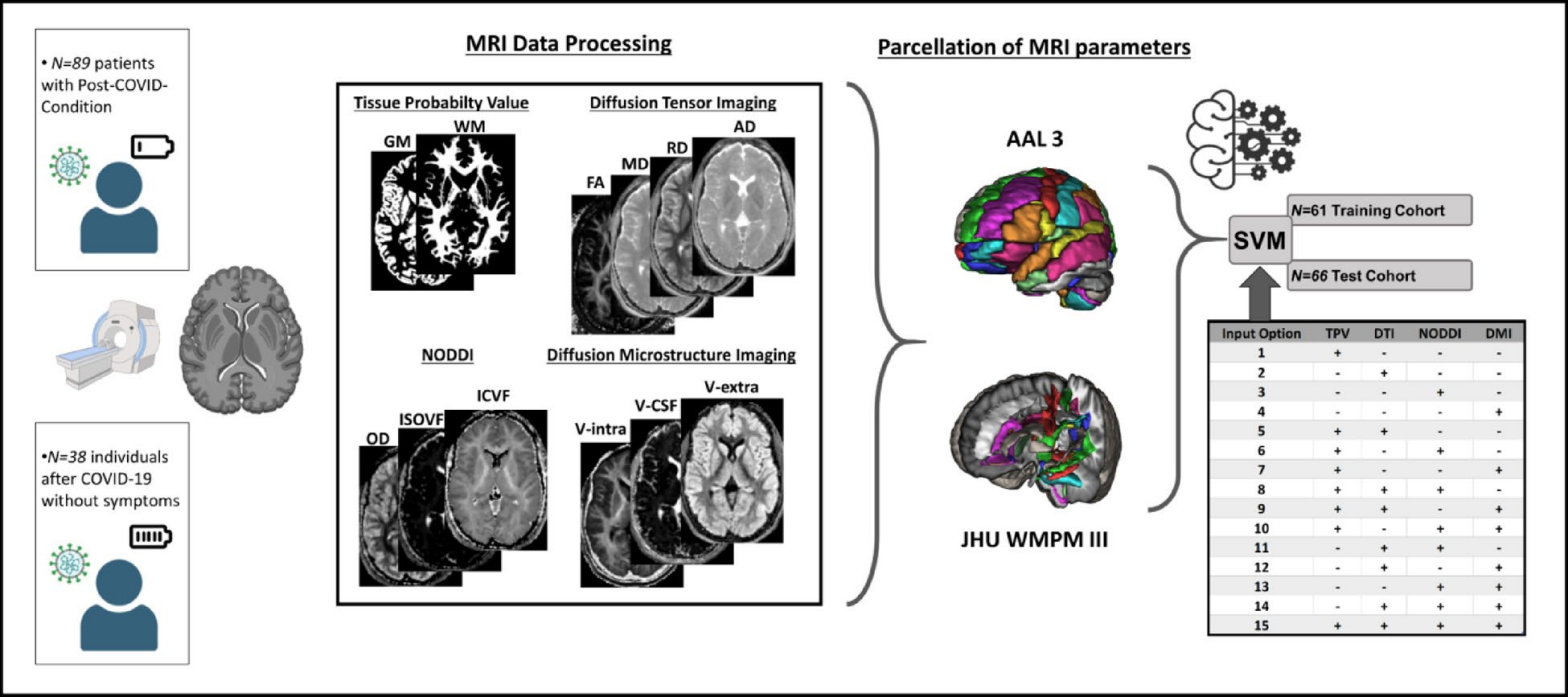
Schematic overview of the study workflow.

An overview of the study workflow is provided in Fig. 1.

### Study participants and clinical outcomes

We report data from a previously published monocentric, prospective cohort of 89 patients (median age: 49 with IQR [23] years; 34/55 males/females), who were admitted to the outpatient clinic of the Department of Neurology and Clinical Neuroscience of the University Hospital Freiburg between June 2020, and October 2024 due to neurocognitive symptoms in the chronic phase after COVID-19 infection^13^. The ethics committee of the Albert-Ludwigs-University Freiburg approved this study (EK 211/20) and all subjects provided written informed consent. The study was conducted in accordance with the Declaration of Helsinki and its later amendments. Inclusion criteria were: (1) reverse transcription polymerase chain reaction (rt-PCR) confirmed SARS-CoV-2 infection; (2) fulfillment of diagnostic criteria for the “Post-COVID-Condition” (PCC) according to the WHO-definition (e.g. > 3 months since onset of acute COVID-19 infection; symptoms lasting for at least two months; relevant impact on everyday functioning)^4^; (3) execution of a cerebral MRI. Exclusion criteria were any pre-existing neurodegenerative disorders and age < 18 years. The “Unimpaired Post-COVID” (UPC)-cohort served as the control group, comprising a collective of 38 subjects (median age: 42 [24] years; 13/25 males/females) in the chronic phase following a PCR-confirmed diagnosis of SARS-CoV-2 infection without persistent subjective complaints. The same exclusion criteria were applied (i.e., any pre-existing neurodegenerative disorders, age < 18 years and artifacts in imaging data), and the examination and measurement methods were identical to the PCC-group. Groups did not significantly differ in age (Mann–Whitney-U, *P* = 0.08) and sex (*X*^*2*^*, P* = 0.60). Patients were examined and surveyed by board-certified (SF, JH) or experienced (> 6 years of training, NS) neurologists. The degree of current disability was graded as follows: 0, no relevant restrictions; 1, relevant restrictions but able to work (i.e. patients who remain able to work but must limit leisure activities, hobbies, and social or family life in order to sustain employment); 2, reduction of work quota necessary; 3, inability to work and/or restriction of daily life activities. Disease severity during the acute stage was scored according to a modified version of the German definitions^14^: 1, no signs of pneumonia; 2, pneumonia, outpatient treatment; 3, pneumonia, inpatient treatment; 4, acute respiratory distress syndrome (ARDS), mechanical ventilation at intensive care unit (ICU). Disease severity was considered to be “mild” in case of outpatient treatment (i.e. 1–2) and as “severe” in patients that required hospitalization (i.e. 3–4). Cognitive functions were assessed with the German version of the Montreal Cognitive Assessment (MoCA version 7.1www.mocatest.org)^15^. The highest possible global MoCA score is 30 with higher scores indicating better performance, the cut-off score for cognitive impairment was defined as < 26^15^. Correction for years of education (YoE) was performed (+ 1 point in case of ≤ 12 YoE). Fatigue was evaluated using the Würzburg Fatigue Inventory in Multiple Sclerosis (WEIMuS)^16^, a self-rating questionnaire for symptoms of physical and cognitive fatigue. In addition, the Geriatric Depression Scale-15 (GDS) was surveyed^17^. Olfaction was assessed using Burghart-Sniffin’-Sticks® (Burghart Messtechnik GmbH, Wedel, Germany; normosmia: 11–12 correctly identified odors; hyposmia: 7–10 correct odors; anosmia: ≤ 6 correct odors)^18^. Ammonium was used to assess trigeminal function.

### Cerebral MRI

#### MRI acquisition

The scanner, device settings and head coil were identical for both groups. MRI was performed with a 3 Tesla scanner (MAGNETOM Prisma, Siemens Healthcare, Erlangen, Germany) with a 64-channel head and neck coil. T1‐weighted (T1w) images were acquired with a three‐dimensional (3D) magnetization‐prepared 180° radio‐frequency pulses and rapid gradient‐echo (MP‐RAGE) sequence (repetition time: 2500 ms, echo time: 2.82 ms, flip angle: 7°, TI = 1100 ms, GRAPPA factor = 2, 1.0 mm^3^ isotropic voxels, 192 contiguous sagittal slices). The diffusion-weighted sequence was acquired with the following parameters: axial orientation, 42 slices, voxel size 1.5 × 1.5 × 3 mm^3^, TR 2800 ms, TE 88 ms, bandwidth 1778 Hz, flip angle 90°, simultaneous multi-band acceleration factor 2, GRAPPA factor 2, 58 diffusion-encoding gradient directions per shell with b-factors 1000 and 2000 s/mm^2^, 15 non-diffusion weighted images (interleaved during diffusion-encoding directions) resulting in overall 131 images.

#### Calculation and extraction of micro- and macrostructural imaging features

The data processing was conducted using our in-house post-processing platform, NORA (www.nora-imaging.org), and was performed in accordance with the previously described methodology^19^. The pre-processing of diffusion-weighted images entailed a denoising step^20^, followed by the correction of Gibbs-ringing artifacts^21^ and upsampling to an isotropic resolution of 1.5 mm^3^. Diffusion microstructural imaging (DMI) metrics were estimated using a Bayesian approach that determines the three components of a white matter-based tissue standard model^10,11^. The first of these is the free water/CSF fraction (V-CSF), which represents the proportion of molecules that move randomly at the distance of their diffusion length (in the range of tenth of micrometers). 2. The volume fraction within neuronal processes/neurites (i.e. axons and dendrites; V-intra) is characterized by almost one-dimensional molecule diffusion due to the presence of tight membrane borders. 3. The volume fraction outside of axons or dendrites (V-extra) is defined by an intermediate constraint to molecule diffusion, representing the cellular compartment and the extracellular matrix. Furthermore, diffusivity parameters were extracted using the accelerated microstructure imaging via convex optimisation (AMICO)-NODDI, a regularized version of the neurite orientation dispersion and density imaging (NODDI) technique that relies on maximum likelihood estimation. The linearisation of fitting procedures (AMICO) enables the rapid processing of data (https://github.com/daducci/AMICO)^22^. In consideration of the parameters provided by the AMICO approach (ISOVF, ICVF, and OD), it can be posited that the isotropic volume fraction (ISOVF) represents the homologue of V-CSF, whereas orientation dispersion (OD) can be seen as a measure of neurite integrity comparable to V-intra. Intracellular volume fraction (ICVF) can be finally seen as a proxy of V-extra. The diffusion tensor imaging (DTI) measures, namely fractional anisotropy (FA), mean diffusivity (MD), radial diffusivity (RD), and axial diffusivity (AD), were obtained from b = 0 and 1000 s/mm^2^. The images were processed using a publicly available open-source toolbox (https://www.uniklinik-freiburg.de/mr-en/research-groups/diffperf/fibertools.html), employing the ordinary log-linear fitting. T1w imaging datasets were automatically segmented into white matter, gray matter and cerebrospinal fluid (CSF) using CAT12 (http://www.neuro.uni-jena.de/cat/) and diffusion magnetic resonance imaging (dMRI) images were coregistered to the T1w images. The validity of the coregistrations between the dMRI images and the tissue probability values derived from the T1-weighted images was manually confirmed. Furthermore, a visual inspection of each individual dMRI map and the CAT12 segmentation was conducted to ensure quality control. The parameter maps of DMI, NODDI, and DTI were separated into gray and white matter using a CAT12-derived tissue probability value (TPV) threshold of 0.4. For this, the TPV provides the probability of a voxel being attributed to gray or white matter. From this, only the gray matter compartment was read for the AAL3 atlas^23^ and only the white matter part for the JHU WMPM III atlas^24^. Gray matter TPV were read from the AAL3 atlas only. From these neuroanatomically established parcellations, the masks used for the extraction of MRI parameters were selected iteratively using the approach described below to avoid selection bias for SVM training, which could be introduced by manually selecting regions.

#### Training of a linear support vector machine (SVM)

As previously described^25^, a linear support vector machine (SVM) was trained and optimized with respect to the area under the receiver operating characteristic curve (AUC-ROC) in a binary classifier for UPC vs. PCC. For this, our cohort was randomly split into a training (n = 43 PCC and n = 18 UPC) and an independent testing subset (n = 46 PCC and n = 20 UPC). A random seed was set in R to automate the splitting process, ensuring a split without human intervention. Subsequent validation confirmed that there were no significant differences in age and sex between the training and testing subsets (*p* > 0.37). The diagnostic performance of the SVM was evaluated in comparison with different inputs, namely TPV, DTI, NODDI, and DMI, both individually and in combination. The SVM was developed using the Python (version 3.8.5) package “Scikit-learn” (version 0.23.2). The atlas-derived microstructural and macrostructural parameters were employed as inputs for the linear SVM. We investigated the diagnostic value of 116 TPV-derived features, 912 features obtained by DTI, 684 by NODDI, and 684 by DMI. To address the considerable disparity in group sizes, the class_weight was set to ‘balanced’. The input parameters were normalized to mean 0 and standard deviation using the Scikit-learn StandardScaler. Prior to the commencement of the training process, the input parameters were sorted in accordance with the principle of maximum marginal diversity^26^. This approach was selected due to the relatively modest group sizes in comparison to the maximum number of input features. In this manner, the normalized and maximum marginal diversity (MMD)-sorted values of a given combination of features are used as input for the linear SVM. To identify the optimal combination of the linear SVM parameter C and the number of the MMD-sorted (descending in diversity) input parameters, a grid search approach was employed, whereby different linear SVMs were trained with C varying between 0.01 and 100 in logarithmic steps and the number of input parameters varying between the top 4–40% in steps of 4%. The resulting models were then fivefold cross-validated and evaluated based on the area under the curve (AUC). To reduce noise and prevent overfitting, the maximum number of input parameters was set to be below 40%. This threshold was obtained by inspecting a graphical depiction of the association of the number of input features with the AUROC in the training cohort.

### Analysis

Statistical analysis was performed using R (version 4.1.0, https://www.R-project.org/) and SPSS, Version 25 (IBM, Ehningen, Germany). No statistical method was used to predetermine sample size. The Shapiro–Wilk test was used to assess the distribution of data. Non-normally distributed data were presented as the median value [inter quartile range] and the non-parametric two-tailed Mann–Witney-U-tests were applied. For comparison of sex and comorbidities, Chi-square test was used. The area under the receiver-operating-characteristics curve was employed to investigate and compare the discriminative power of the different SVM inputs. The significance threshold was set to *p* < 0.05.

## Results

### Demographic and clinical characteristics

We included MRI data from a previously characterized cohort of 89 patients (median age 49 years; IQR [23] years; range: 19–72 years; 55 females) who fulfilled the WHO diagnostic criteria for Post-COVID-condition (PCC group). An overview of demographic and patient characteristics is shown in Table 1**,** and details are provided in Supplementary Table 1 or Hosp et al.^13^. Although neurological examinations revealed no focal deficits, patients complained of impaired attention and memory (100%), fatigue (96%), impaired ability to multitask (97%), and word-finding difficulties (89%). 76 patients (85%) exhibited a mild course of acute SARS-CoV-2 infection, with no requirement for hospitalization. With regard to comorbidities, no significant difference between the PCC and UPC groups was observed (*p* > 0.07). However, given the relatively small size of the UPC group, the statistical power to detect differences is limited, and some of the observed numerical trends (e.g., higher prevalence of obesity and obstructive sleep apnea in the PCC group) may still be clinically relevant and could reach significance in larger cohorts.

**Table 1:** Demographics and comorbidities of study participants.

	Post-COVID-condition (PCC; n = 89)	Unimpaired post-COVID (UPC; n = 38)	
Demographic data	n (%) or median [IQR]; range	(%) or median [IQR]; range	*P* value
Age (years)	49 [23]; 19 to 72	42 [24]; 25 to 62	0.08^1^
Sex (male / female)	34 (38) / 55 (62)	13 (34) / 25 (66)	0.60^2^
Δ positive PCR—cMRI (days)	254 [209]; 90 to 710	227 [443]; 145 to 943	0.63^1^
Comorbidities	n (%)	n (%)	*P* value^2^
Obesity	12 (14%)	3 (8%)	0.07
Asthma/COPD	8 (9%)	1 (3%)	0.20
Atrial fibrillation	1 (1%)	0 (0%)	0.51
Chronic kidney diesease	1 (1%)	0 (0%)	0.51
Coronary heart disease	3 (3%)	1 (3%)	0.83
Diabetes	5 (6%)	1 (3%)	0.47
History of depression	9 (10%)	2 (5%)	0.37
History of ischemic stroke	2 (2%)	0 (0%)	0.35
Arterial hypertension	19 (21%)	4 (10%)	0.15
Hypothyreodism	9 (8%)	6 (16%)	0.36
Malignancy	3 (3%)	0 (0%)	0.25
Migraine	10 (11%)	1 (3%)	0.11
Obstructive sleep apnoea	7 (8%)	0 (0%)	0.08
Peripheral arterial occlusive disease	1 (1%)	0 (0%)	0.51
Restless legs syndrome	2 (2%)	0 (0%)	0.35

^1^Mann–Whitney-U test; ^2^*X*^*2*^-test.

### Evaluation of conventional MRI

As previously reported, six patients with PCC exhibited mild microangiopathic white matter changes corresponding to Fazekas 1^27^. In one patient (a 39 years-old female), a small, primarily gliotic lesion was identified in the right basal ganglia. In another patient (66 years-old male), an occipital cortical defect was identified, which was deemed to be a probable post-ischemic lesion. In a third patient (62 years-old male), slight T2 signal elevations were observed bilaterally in the globus pallidum, without any correlation to other MRI sequences. No further structural changes, signs of atrophy or any evidence of inflammation (e.g. leptomeningeal enhancement) were identified. Within the UPC group, symmetrical hyperintense T2 signals of unknown origin were observed in one patient (39 year-old male) and slight microangiopathic lesions were noted in two patients (both Fazekas 1).

### Comparison of input combinations and most discriminative regions

Upon comparison of the different input combinations in the testset, we found the highest AUROC for (a) DMI and NODDI (0.95), (b) DMI, DTI and NODDI (0.94), and (c) DMI, NODDI and TPV (0.94). Of note, TPV alone reached an AUROC of 0.59 only and DTI alone only 0.78. Further details are provided in Table 2.

**Table 2:** Comparison of different SVM-input combinations.

Input combination				SVM performance						
DMI	DTI	NODDI	TPV	AUROC	FP	FN	Specificity	Sensitivity	Precision	F1-Score
+				0.83	13	5	0.75 (0.53–0.89)	0.72 (0.57–0.83)	0.54 (0.36–0.70)	0.63
+			+	0.88	7	6	0.70 (0.48–0.85)	0.85 (0.72–0.92)	0.67 (0.45–0.83)	0.68
** + **		** + **		0.95	3	3	0.85 (0.64–0.95)	0.94 (0.82–0.98)	0.85 (0.64–0.95)	0.85
** + **		** + **	** + **	0.94	3	4	0.80 (0.58–0.92)	0.93 (0.82–0.98)	0.84 (0.62–0.94)	0.82
** + **	** + **			0.85	4	9	0.55 (0.34–0.74)	0.91 (0.80–0.97)	0.73 (0.48–0.89)	0.63
** + **	** + **		** + **	0.87	6	7	0.65 (0.43–0.82)	0.87 (0.74–0.94)	0.68 (0.46–0.85)	0.67
** + **	** + **	** + **		0.94	3	5	0.75 (0.53–0.89)	0.94 (0.82–0.98)	0.83 (0.61–0.94)	0.79
** + **	** + **	** + **	** + **	0.93	4	3	0.85 (0.64–0.95)	0.91 (0.80–0.97)	0.81 (0.60–0.92)	0.83
			** + **	0.59	24	8	0.60 (0.39–0.78)	0.48 (0.34–0.62)	0.33 (0.20–0.50)	0.43
		** + **		0.89	6	5	0.75 (0.53–0.89)	0.87 (0.74–0.94)	0.71 (0.50–0.86)	0.73
		** + **	** + **	0.87	6	5	0.75 (0.53–0.89)	0.87 (0.74–0.94)	0.71 (0.50–0.86)	0.73
	** + **			0.78	11	6	0.70 (0.48–0.85)	0.76 (0.62–0.86)	0.56 (0.37–0.73)	0.62
	** + **		** + **	0.77	14	2	0.90 (0.70–0.97)	0.70 (0.55–0.81)	0.56 (0.39–0.72)	0.69
	** + **	** + **		0.89	8	4	0.80 (0.58–0.92)	0.83 (0.69–0.91)	0.67 (0.47–0.82)	0.73
	** + **	** + **	** + **	0.91	6	5	0.75 (0.53–0.89)	0.87 (0.74–0.94)	0.71 (0.50–0.86)	0.73

AUROC, area under the ROC curve; DMI, diffusion microstructural imaging; DTI, diffusion tensor imaging; FP, number of false positives; FN, number of false negatives; NODDI, neurite orientation dispersion and density imaging; TPV, tissue probability value.

For the best-performing combination of DMI and NODDI, the maximum marginal diversity algorithm revealed a high diversity of the parameters, especially for frontal, frontobasal, temporal and infratentorial regions as given in Supplementary Table 2.

**Fig. 2:**
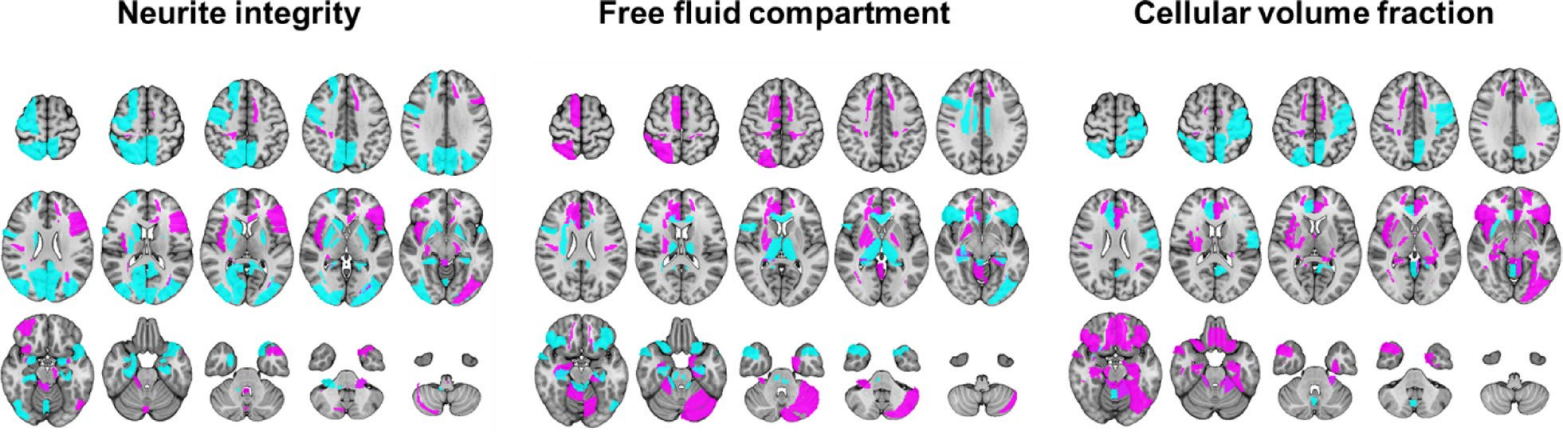
Regions with high coefficients of the support vector machine for discriminating post-COVID-Condition from unimpaired-post-COVID for the best performing input combination of diffusion-microstructure-imaging (DMI) and neurite orientation dispersion and density imaging (NODDI). Atlas-defined regions with negative coefficients are shown in cyan and positive coefficients in magenta.

To better understand the distinction between PCC patients and UPC individuals, we first extracted the atlas-based DMI/NODDI-derived diffusivity parameters from the training cohort that exhibited the highest discriminatory power, as indicated by their support vector machine (SVM) coefficients. A threshold of ± 0.15 was applied for feature selection (see Supplementary Table 3). To gain further insight into the underlying neurobiological processes, we categorized the selected parameters from DMI and NODDI into three biologically relevant groups: **Neurite integrity**, combining intra-axonal volume fraction (V-intra, from DMI) and orientation dispersion (OD, from NODDI); **Free fluid compartment**, combining cerebrospinal fluid volume fraction (V-CSF, from DMI) and isotropic volume fraction (ISOVF, from NODDI); and **Cellular volume fraction**, combining extra-axonal volume fraction (V-extra, from DMI) and intracellular volume fraction (ICVF, from NODDI). The spatial distribution of both positive and negative SVM coefficients for each feature category is visualized in Fig. 2.

With regard to the **“neurite integrity”** category, we observed increased metrics primarily in the left occipital cortex, infratentorial regions, right insula, left frontal operculum, and right orbitofrontal cortex. In contrast, decreased neurite integrity was evident across widespread regions, including the basal ganglia, limbic system, and the fronto-parieto-occipital cortex. In the **“free fluid compartment”**, elevated values were detected in the cerebellum, frontal white matter, cingulum, and basal ganglia, whereas reductions were observed in the thalamus, brainstem, temporal poles, corpus callosum, and frontal operculum. Regarding the **“cellular volume fraction”** category, increased values were found in the orbitofrontal cortex, limbic regions, left occipital cortex, left insula, and frontal white matter. Decreased values were noted in the left paracentral lobule, right parietal cortex, and the vermis.

In summary, the findings reveal a spatially widespread and directionally heterogeneous pattern of alterations across both white and gray matter compartments. This underscores not only the value but also the necessity of applying artificial intelligence techniques to capture such complex patterns for diagnostic purposes and for discriminating between individuals with PCC and UPC.

## Discussion

In this study, we employed artificial intelligence to develop an imaging-based biomarker-assisted diagnosis of PCC on a single-patient level. We trained a linear SVM to distinguish between PCC patients and asymptomatic individuals who had been infected with SARS-CoV-2 in the past. Upon comparing the diagnostic value of macro- and microstructural parameters, best discrimination between groups was found for multicompartimental microstructural approaches, while macrostructural information did not contribute to diagnostic accuracy. The optimal algorithm achieved an AUROC value of 0.95 with a sensitivity of 94% and a specificity of 85%. The atlas regions with the highest discriminatory power include both gray matter (including multiple cortical areas, putamen, and left thalamus) and white matter (including corpus callosum and frontal white matter).

Given the previous detection of macrostructural atrophy in patients with PCC^6,7^, it is unexpected that TPV, as an input factor, does not contribute to the discrimination from the UPC group. It is crucial to acknowledge that the aforementioned studies employed control groups composed solely of individuals without a history of previous SARS-CoV-2 infection, i.e. who were healthy. Nevertheless, there is compelling evidence that SARS-CoV-2 infection can induce structural alterations in the brain that are not contingent on PCC. A comprehensive longitudinal study from the UK Biobank examined MRI data of participants before and 4–5 months after the onset of SARS-CoV-2 infection^28^. The results demonstrated alterations in macrostructure, as well as diffusion-based indices: A decrease in global brain volume was observed, accompanied by a reduction in cortical thickness in several regions, including the parahippocampal gyrus, anterior cingulate cortex, temporal pole, and the left orbitofrontal cortex, insula, and supramarginal gyrus. Moreover, an increase in diffusion indices within the limbic regions (anterior cingulate, hippocampal, parahippocampal, and orbito-frontal cortex) and the striatum indicated microstructural changes in these regions. The limited clinical characterization of the cohort precluded the drawing of any conclusions regarding the prevalence of PCC. However, if one assumes that a maximum of 10% of those infected develop PCC^1,2^, it must be expected that the aforementioned SARS-CoV-2-triggered structural changes may also occur independently of PCC disease. This hypothesis was corroborated by a recent publication on the same cohort we employed in this study^13^. In the previous study, the patients with PCC were compared not only with the UPC group but also with a control group of healthy subjects who had never contracted COVID-19. With regard to microstructure, both the PCC and UPC groups exhibited a volume shift from the membrane-enclosed compartment into the free-water compartment within the (sub-)cortical gray matter. In contrast, an increase of the membrane-enclosed compartment was present within the corpus callosum, internal capsule, cerebellum, and brainstem. Nevertheless, the PCC and UPC groups could be distinguished by their disparate emphasis on the aforementioned pattern. However, the partial similarity of patterns with respect to microstructural changes explains that, despite high sensitivity, only a specificity of 85% could be achieved. In conclusion, an image-based diagnosis of PCC must be based on a control-collective that has also contracted COVID-19 in order to accurately detect PCC-specific changes. In this context, microstructural parameters are of particular importance. Noteworthy, we found a substantially superior performance in the biophysically motivated multicompartimental techniques NODDI and DMI upon comparison with single compartment DTI. We attribute this to the superior approximation of the actual cerebral microstructure in these approaches which aligns with other studies^25,29^. The findings in conventional MRI in individual patients with PCC are non-specific and cannot account for the overall microstructural alterations present in the PCC cohort.

To better characterize the distinction between PCC and UPC, we identified the most relevant discriminatory features and grouped them into the overarching categories of neurite integrity, free fluid, and cellular volume fraction, based on microstructural metrics derived from DMI and NODDI. This analysis revealed a spatially widespread, yet partially overlapping pattern of alterations across these domains (Fig. 2). The distribution of changes suggests that multiple brain networks may be affected in PCC, potentially contributing to the broad and heterogeneous symptom spectrum commonly reported in affected individuals^3^. Importantly, alterations were not confined to a single domain or anatomical region, but encompassed cortical, subcortical, and cerebellar areas across all categories. These spatial patterns are broadly in line with prior findings of thalamic and subcortical involvement in PCC, including microstructural thalamic changes^7^, widespread cortical affection^13,28^, and white matter alterations, all linked to fatigue or cognitive symptoms^6,8,13^.

Although the exact biological mechanisms remain to be elucidated, the concurrent involvement of multiple tissue compartments may reflect a complex and multifactorial pathophysiology. However, the cellular and histopathological basis of the (micro)structural changes in PCC detected by imaging is not well understood. Histopathologic studies of patients who died of severe COVID-19 infections in the acute phase have provided a relatively clear picture of blood–brain barrier disruption, specific activation of perivascular lymphocytes, and transduction of the inflammatory signal into the parenchyma with activation of astrocytes, microglia, and formation of microglial nodules^30–33^. However, histopathologic data from the late phase of COVID-19 infection are rare. Examinations of the medulla oblongata of patients who died of sudden cardiac death even months after SARS-CoV-2 infection revealed not only persistent T-cell activation^31^, but also the presence of a SARS-CoV-2-specific innate immune scar, as evidenced by the persistence of microglial nodules^33^. With respect to neurite integrity, an increase may reflect enhanced structural coupling or a neuroplastic response^34^, whereas a decrease is more likely indicative of axonal loss and reduced connectivity between brain regions^35^. In our analysis, we observed a predominant decrease in neurite integrity, with pronounced involvement of the basal ganglia, limbic system, and fronto-parieto-occipital cortex. Regarding the free fluid category, an increase may be attributed to blood–brain barrier dysfunction or cerebral edema^19^, but could also reflect secondary atrophic processes^36^. Conversely, a decrease in free fluid may suggest gliotic transformation or inflammatory infiltration^37^. In the current analysis, we found a predominant increase in free fluid, particularly affecting the cerebellum, frontal white matter, cingulum, and basal ganglia. Lastly, an increase in cellular volume fraction may indicate gliotic changes or the presence of an inflammatory infiltrate^37^, whereas a decrease could be suggestive of an underlying neurodegenerative process^36^. Our findings revealed a predominant increase in cellular volume fraction, especially in the orbitofrontal cortex, limbic regions, left occipital cortex, left insula, and frontal white matter. Further studies are warranted to validate these findings and to explore their relationship with histopathological changes and specific clinical phenotypes or symptom clusters in PCC.

In light of the favorable diagnostic accuracy of our SVM methodology, imaging-supported diagnosis of PCC appears to be a viable prospect. As our study employs monocentric data, the subsequent step would be to validate the SVM approach to a prospective multicenter cohort. The SVM algorithm developed in our cohort would be immediately applicable for this purpose. However, retraining using a multicenter control group (analogous to our UPC collective) would serve as an additional measure to validate our approach. In addition to our structural and diffusion imaging-based strategy, the incorporation of other modalities for the objective diagnosis of PCC would be advantageous. While the sensitivity of the proposed approach can be deemed sufficient for clinical application, the specificity of 85% should be supplemented with other imaging parameters or biomarkers. In the serum of a patient with severe SARS-CoV-2 infection, elevations in glial fibrillary acidic protein (GFAP), a marker of astrocyte activation, and neurofilament light chain (NfL), a marker of neuronal damage, were observed during the acute phase^38^. However, these values return to normal levels over time^39^ and are not increased in patients with PCC^40^. Apart from this, high-throughput approaches have revealed group-level differences between PCC patients and controls with regard to circulating immune cell populations, antibody levels against SARS-CoV-2, EBV, and VZV, and reduced cortisol and serotonin levels^41,42^. Furthermore, a protein signature in the blood was identified, characterized by increased complement activation and thromboinflammation. This signature included activated platelets and markers of red blood cell lysis^43^. To date, no attempt has been made to utilize such a multi-parametric serological approach to facilitate diagnosis at the individual patient level. Moreover, the creation of a comprehensive data set that incorporates serological analyses, cerebral MR imaging, and clinical characterization through questionnaires and neurocognitive diagnostics would be beneficial for facilitating a biomarker-based diagnosis of PCC.

Several limitations of our study should be acknowledged. First, although our findings demonstrate a correlation between neuroimaging parameters and clinical symptoms of PCC, causality cannot be established. Longitudinal studies incorporating detailed clinical assessments are needed to better understand the temporal dynamics and persistence of PCC-related symptoms and to clarify whether observed microstructural alterations are a cause or consequence of clinical impairment. Second, this study employed a monocentric design, which may limit generalizability. While our control group (UPC) provides a robust clinical comparison, external validation through multicenter datasets, ideally using harmonized imaging protocols and similarly defined control groups, is essential to confirm the reproducibility and applicability of our approach. Here, a larger group size of UPC in the testing dataset might have improved stability of the specificity. Third, the use of advanced diffusion imaging based on multi-shell protocols necessitated scanning at 3 T field strength to ensure sufficient data quality. Although such imaging systems are available at most academic radiology departments, this requirement may limit immediate broader clinical adoption, particularly in non-academic settings. A further limitation arises from the relatively small size of the UPC training cohort (n = 18). Although class balancing and cross-validation were applied, the stability of the selected discriminative features may be strongly influenced by the specific composition of this subgroup. Moreover, even though the headline AUROC of 0.95 indicates excellent discriminatory potential, the wide confidence intervals around key performance metrics highlight substantial uncertainty. For the best-performing model, specificity was 85%, but the 95% confidence interval ranged from 0.64 to 0.95, and precision showed the same range. This largely attributable to the small size of the UPC test group (n = 20) and underscores the need for replication in larger, multicentric datasets to obtain more stable and clinically reliable estimates.

In conclusion, our processing approach allowed for the reliable differentiation between PCC patients and UPC participants with high sensitivity, while specificity remained moderate. This highlights both the potential of microstructural MRI as a diagnostic tool on a single patient level, but also the necessity of further validation and multimodal approaches before clinical translation.

## Supplementary Information

Below is the link to the electronic supplementary material.

Supplementary Material 1

## Data Availability

Data is available from the corresponding author (JAH) upon reasonable request and approval of the local ethics committee.
