# Supplementary material for: Cerebral microstructural alterations as an imaging biomarker for Post-COVID-condition
Source: Sci Rep. 2025 Sep 12;15:32483. doi: 10.1038/s41598-025-18962-3 (PMC12432129; doi:10.1038/s41598-025-18962-3)
Supplement: Supplementary file 1 — Supplementary Material 1 [file 41598_2025_18962_MOESM1_ESM.docx]

**Supplement**

| **Supplementary Table 1: Clinical characteristics of study participants (adapted from Hosp et al., 2024**^1^**)** | | | |
| --- | --- | --- | --- |
|  | **Post-COVID-Condition**  **(PCC; n = 89)** | **Unimpaired Post-COVID**  **(UPC; n = 38)** |  |
| Clinical readouts | median [IQR]; range; n outside of norm (%) | median [IQR]; range; n outside of norm (%) |  |
| Disease severity score^2^ | n (%) | n (%) |  |
| 1  2  3  4 | 42 (47%)  34 (38%)  11 (13%)  2 (2%) | 25 (65%)  12 (32%)  1 (3%)  0 (0%) |  |
| Grading of current disability3 | n (%) | n (%) |  |
| 0  1  2  3 | 0 (0%)  42 (47%)  11 (13%)  36 (40%) | 38 (100%)  0 (0%)  0 (0%)  0 (0%) |  |
| Current neurological symptoms | n (%) | n (%) |  |
| Impaired attention  Memory impairment  Impaired multi-tasking  Word-finding difficulties  Fatigue | 89 (100%)  89 (100%)  86 (97%)  79 (89%)  85 (96%) | 0 (0%)  0 (0%)  0 (0%)  0 (0%)  0 (0%) |  |
| Clinical readouts | score | score | P-value^4^ |
| MoCA sum score (corrected for years of education; norm ≥ 26/30)    Correct perception of smell (norm ≥ 11/12)    Würzburg Fatigue Inventory in Multiple Sclerosis: sum score (WEIMuS; norm: ≤ 33/68)^5^  Geriatric Depression Scale (GDS-15; norm: ≤ 7/15)^5^ | 26 [4]; 18 to 30; 36 (41%)    9 [4]; 0 to 12; 66 (74%)  43 [17]; 4 to 65; 65 (78%)    4 [5]; 1 to 13; 16 (19%) | 27 [3]; 23 to 30; 7 (18%)  11 [2]; 7 to 12; 16 (42%)    7 [18]; 0 to 48; 3 (8%)  1 [2]; 0 to 9; 1 (3%) | 0.003    < 0.001    < 0.001  < 0.001 |
| ^1^Hosp, J. A. *et al.* Cerebral microstructural alterations in Post-COVID-condition are related to cognitive impairment,  olfactory dysfunction and fatigue. *Nat Commun* **15**, 4256 (2024). ^2^ Disease severity score: (1) no pneumonia; (2)  pneumonia, outpatient treatment; (3) pneumonia, inpatient treatment; (4) ARDS, endotracheal ventilation at ICU; ^3^  Grading of current disability: (0) no relevant restrictions; (1) relevant restrictions of daily life activities but able to  work; (2) reduction of work quota necessary; (3) inability to work and restriction of daily life activities; ^4^ Mann  Whitney-U test; ^5^ data available only for n = 88 patients. | | | |

| **Supplementary Table 2: Atlas regions with high variance in the training cohort as derived from the maximum marginal diversity algorithm** | | | |
| --- | --- | --- | --- |
| Atlas | Region | Feature | Variance |
| AAL | Vermis_3 | V-extra | 0.31553773 |
| AAL | Cerebelum_3_R | V-extra | 0.23984748 |
| AAL | Hippocampus_R | V-intra | 0.23902991 |
| AAL | Vermis_3 | V-CSF | 0.23636462 |
| AAL | Vermis_1_2 | V-CSF | 0.23588727 |
| JHU | MIDDLE_TEMPORAL__WM_left | ICVF | 0.2354118 |
| JHU | Genu_of_corpus_callosum_left | V-CSF | 0.23240806 |
| AAL | Temporal_Pole_Mid_R | V-extra | 0.22974543 |
| AAL | Frontal_Inf_Oper_R | V-extra | 0.22394205 |
| JHU | ENTORHINAL_AREA_right | V-extra | 0.22281888 |
| JHU | PRE_CUNEUS__WM_left | V-extra | 0.22026398 |
| AAL | Temporal_Pole_Mid_R | V-CSF | 0.21981969 |
| JHU | MIDDLE_TEMPORAL_WM_right | V-extra | 0.21063111 |
| AAL | Frontal_Inf_Oper_R | V-CSF | 0.20892495 |
| JHU | PRE_CUNEUS_WM_right | ICVF | 0.20877402 |
| JHU | PONS_left | ICVF | 0.20795298 |
| AAL | Temporal_Mid_L | V-extra | 0.2066709 |
| AAL | Frontal_Inf_Orb_R | V-extra | 0.20453456 |
| AAL | Frontal_Inf_Tri_L | V-extra | 0.20321988 |
| AAL | Cerebelum_3_L | V-extra | 0.20213179 |
| AAL | Cerebelum_10_L | ISOVF | 0.20165631 |
| AAL | Cerebelum_10_R | V-CSF | 0.19957624 |
| AAL | Vermis_1_2 | V-intra | 0.19955255 |
| AAL | ParaHippocampal_R | V-extra | 0.1991128 |
| JHU | PRECENTRAL_WM_right | OD | 0.19888763 |
| AAL | Vermis_10 | V-intra | 0.19787806 |
| AAL | Cerebelum_3_L | ISOVF | 0.19725999 |
| JHU | Posterior_limb_of_internal_capsule_right | ICVF | 0.19475316 |
| JHU | HIPPOCAMPUS | ICVF | 0.19198306 |
| JHU | SUPRAMARGINAL_WM_right | ICVF | 0.19146822 |
| AAL | Caudate_L | V-extra | 0.19140804 |
| AAL | ParaHippocampal_R | V-CSF | 0.19122751 |
| AAL | Rectus_L | V-CSF | 0.19115731 |
| AAL | Frontal_Inf_Oper_L | ISOVF | 0.19023754 |
| AAL | Cerebelum_Crus1_R | V-CSF | 0.1899907 |
| AAL | Temporal_Pole_Sup_L | OD | 0.18899119 |
| JHU | Body_of_corpus_callosum_left | OD | 0.18631306 |
| AAL | Amygdala_R | V-extra | 0.18593712 |
| AAL | Postcentral_L | ISOVF | 0.18535569 |
| JHU | SUPRAMARGINAL__WM_left | V-extra | 0.18471377 |
| AAL | Supp_Motor_Area_R | V-CSF | 0.18469744 |
| AAL | Vermis_4_5 | ICVF | 0.18452086 |
| JHU | Posterior_limb_of_internal_capsule_right | V-intra | 0.18433844 |
| AAL | Insula_R | V-intra | 0.1831272 |
| AAL | Cerebelum_Crus1_R | ISOVF | 0.1830405 |
| AAL | Insula_R | OD | 0.18228942 |
| JHU | HIPPOCAMPUS_right | V-CSF | 0.18184353 |
| JHU | Cingulum__hippocampus__right | V-extra | 0.18108469 |
| JHU | MIDDLE_FRONTAL__WM_left | V-extra | 0.17934505 |
| JHU | MIDDLE_TEMPORAL__WM_left | V-intra | 0.17840408 |

| **Supplementary Table 3: Atlas regions with high diagnostic value in the training cohort as derived from the support vector machine** | | | | |
| --- | --- | --- | --- | --- |
| **Atlas** | **Region** | **FeatureCategory** | **Feature** | **SVM-Coeffcient** |
| AAL | Occipital_Inf_L | neurite | OD | 0.0404 |
| AAL | Vermis_1_2 | neurite | V-intra | 0.0339 |
| AAL | Vermis_3 | neurite | V-intra | 0.0299 |
| JHU | Genu_of_corpus_callosum_L | neurite | OD | 0.0298 |
| JHU | Cerebral_peduncle_L | neurite | OD | 0.0282 |
| JHU | POSTCENTRAL_WM_R | neurite | V-intra | 0.0270 |
| AAL | Cerebelum_3_R | neurite | V-intra | 0.0260 |
| AAL | Cerebelum_10_L | neurite | V-intra | 0.0259 |
| JHU | Uncinate_fasciculus_L | neurite | OD | 0.0253 |
| AAL | Frontal_Mid_Orb_L | neurite | V-intra | 0.0238 |
| JHU | PONS_L | neurite | OD | 0.0234 |
| JHU | MEDULLA_L | neurite | OD | 0.0232 |
| AAL | Vermis_7 | neurite | OD | 0.0231 |
| JHU | Uncinate_fasciculus_R | neurite | OD | 0.0207 |
| AAL | Cerebelum_7b_R | neurite | V-intra | 0.0206 |
| JHU | SUPERIOR_FRONTAL_WM_L | neurite | OD | 0.0203 |
| JHU | MIDDLE_OCCIPITAL_WM_L | neurite | OD | 0.0186 |
| JHU | Fornix_cres__Stria_terminalis__R | neurite | V-intra | 0.0172 |
| AAL | Insula_R | neurite | OD | 0.0171 |
| AAL | Frontal_Inf_Oper_L | neurite | V-intra | 0.0167 |
| AAL | Temporal_Pole_Mid_L | neurite | V-intra | 0.0166 |
| AAL | Vermis_10 | neurite | V-intra | 0.0158 |
| AAL | Frontal_Inf_Tri_L | neurite | V-intra | 0.0157 |
| JHU | Tapatum_R | neurite | OD | 0.0152 |
| JHU | Inferior_cerebellar_peduncle_R | neurite | OD | -0.0154 |
| AAL | ParaHippocampal_R | neurite | V-intra | -0.0155 |
| JHU | Superior_fronto_occipital_fasciculus__R | neurite | OD | -0.0157 |
| AAL | Cuneus_R | neurite | OD | -0.0162 |
| JHU | PONS_L | neurite | V-intra | -0.0179 |
| AAL | Cuneus_L | neurite | V-intra | -0.0183 |
| JHU | INFERIOR_OCCIPITAL_WM_R | neurite | OD | -0.0184 |
| AAL | Cerebelum_10_R | neurite | OD | -0.0188 |
| JHU | HIPPOCAMPUS | neurite | OD | -0.0188 |
| JHU | ANGULAR_WM_R | neurite | V-intra | -0.0203 |
| AAL | Calcarine_R | neurite | OD | -0.0205 |
| AAL | Vermis_6 | neurite | OD | -0.0231 |
| AAL | Precuneus_R | neurite | OD | -0.0233 |
| AAL | Cingulum_Post_L | neurite | V-intra | -0.0238 |
| JHU | Uncinate_fasciculus_R | neurite | V-intra | -0.0244 |
| AAL | Frontal_Sup_R | neurite | OD | -0.0248 |
| AAL | Amygdala_R | neurite | V-intra | -0.0248 |
| AAL | Precuneus_L | neurite | OD | -0.0249 |
| JHU | SUPERIOR_OCCIPITAL_WM_R | neurite | OD | -0.0249 |
| AAL | Temporal_Pole_Sup_L | neurite | OD | -0.0250 |
| JHU | Posterior_limb_of_internal_capsule_R | neurite | V-intra | -0.0268 |
| AAL | Parietal_Sup_R | neurite | OD | -0.0283 |
| AAL | Occipital_Mid_L | neurite | OD | -0.0292 |
| AAL | Cerebelum_10_L | neurite | OD | -0.0296 |
| AAL | Vermis_3 | neurite | OD | -0.0297 |
| AAL | Occipital_Inf_R | neurite | V-intra | -0.0305 |
| AAL | Occipital_Mid_R | neurite | OD | -0.0325 |
| JHU | PRE_CUNEUS__WM_L | neurite | OD | -0.0330 |
| AAL | Caudate_R | neurite | OD | -0.0337 |
| JHU | PUTAMEN_R | neurite | OD | -0.0387 |
| AAL | Amygdala_L | neurite | OD | -0.0404 |
| JHU | PUTAMEN_L | neurite | OD | -0.0410 |
| AAL | Precentral_R | neurite | OD | -0.0505 |
| JHU | Uncinate_fasciculus_R | cellular | V-extra | 0.0406 |
| JHU | POSTCENTRAL_WM_R | cellular | ICVF | 0.0373 |
| AAL | Occipital_Inf_L | cellular | V-extra | 0.0354 |
| JHU | LINGUAL_WM_L | cellular | V-extra | 0.0336 |
| AAL | Rectus_L | cellular | ICVF | 0.0332 |
| JHU | HIPPOCAMPUS_R | cellular | V-extra | 0.0327 |
| JHU | Inferior_fronto_occipital_fasciculus_L | cellular | V-extra | 0.0282 |
| JHU | INFERIOR_OCCIPITAL_WM_R | cellular | ICVF | 0.0281 |
| AAL | Fusiform_L | cellular | ICVF | 0.0278 |
| JHU | Posterior_limb_of_internal_capsule_R | cellular | V-extra | 0.0260 |
| JHU | Cingulum__hippocampus__R | cellular | V-extra | 0.0255 |
| JHU | Fornix__column_and_body__R | cellular | V-extra | 0.0248 |
| AAL | Frontal_Inf_Orb_L | cellular | V-extra | 0.0246 |
| JHU | GLOBUS_PALLIDUS_L | cellular | ICVF | 0.0236 |
| JHU | Cerebral_peduncle_R | cellular | V-extra | 0.0235 |
| AAL | Insula_R | cellular | ICVF | 0.0221 |
| AAL | Rectus_R | cellular | ICVF | 0.0218 |
| JHU | HIPPOCAMPUS | cellular | V-extra | 0.0216 |
| JHU | SUPERIOR_FRONTAL_WM_L | cellular | ICVF | 0.0215 |
| AAL | Cingulum_Ant_L | cellular | ICVF | 0.0192 |
| JHU | SUPERIOR_FRONTAL__WM_R | cellular | ICVF | 0.0189 |
| JHU | INFERIOR_TEMPORAL_WM_R | cellular | V-extra | 0.0189 |
| AAL | Vermis_3 | cellular | V-extra | 0.0183 |
| AAL | Vermis_1_2 | cellular | V-extra | 0.0171 |
| JHU | External_capsule_R | cellular | V-extra | 0.0170 |
| AAL | Temporal_Pole_Mid_R | cellular | V-extra | 0.0170 |
| JHU | Fornix_cres__Stria_terminalis__R | cellular | V-extra | 0.0170 |
| AAL | Cerebelum_4_5_L | cellular | ICVF | 0.0167 |
| AAL | Frontal_Inf_Orb_R | cellular | V-extra | 0.0166 |
| AAL | Hippocampus_L | cellular | ICVF | 0.0155 |
| AAL | Frontal_Sup_Orb_L | cellular | V-extra | 0.0153 |
| AAL | Cerebelum_3_L | cellular | V-extra | 0.0153 |
| JHU | Retrolenticular_part_of_internal_capsule_L | cellular | V-extra | 0.0152 |
| AAL | Cerebelum_3_R | cellular | V-extra | 0.0152 |
| JHU | Fornix_cres__Stria_terminalis__R | cellular | ICVF | 0.0151 |
| JHU | ANGULAR__WM_L | cellular | ICVF | 0.0151 |
| JHU | FUSIFORM_WM_L | cellular | V-extra | -0.0154 |
| AAL | Cerebelum_4_5_L | cellular | V-extra | -0.0157 |
| JHU | PRE_CUNEUS__WM_L | cellular | V-extra | -0.0166 |
| AAL | Cingulum_Ant_R | cellular | V-extra | -0.0171 |
| AAL | Cerebelum_3_R | cellular | ICVF | -0.0185 |
| AAL | Precentral_L | cellular | ICVF | -0.0186 |
| AAL | Vermis_8 | cellular | V-extra | -0.0205 |
| AAL | Vermis_4_5 | cellular | V-extra | -0.0212 |
| AAL | Precuneus_L | cellular | ICVF | -0.0220 |
| JHU | Inferior_fronto_occipital_fasciculus_R | cellular | ICVF | -0.0226 |
| JHU | MIDDLE_FRONTAL__WM_L | cellular | V-extra | -0.0262 |
| AAL | Parietal_Sup_R | cellular | ICVF | -0.0276 |
| AAL | Postcentral_L | cellular | ICVF | -0.0276 |
| JHU | POSTCENTRAL_WM_R | cellular | V-extra | -0.0311 |
| JHU | INFERIOR_OCCIPITAL_WM_R | freefluid | ISOVF | 0.0474 |
| JHU | FUSIFORM_WM_L | freefluid | ISOVF | 0.0445 |
| JHU | PUTAMEN_R | freefluid | ISOVF | 0.0378 |
| JHU | POSTCENTRAL_WM_R | freefluid | ISOVF | 0.0346 |
| JHU | MIDDLE_FRONTO_ORBITAL_WM_R | freefluid | ISOVF | 0.0313 |
| JHU | POSTCENTRAL__WM_L | freefluid | ISOVF | 0.0288 |
| JHU | SUPERIOR_FRONTAL__WM_R | freefluid | ISOVF | 0.0276 |
| AAL | ParaHippocampal_L | freefluid | ISOVF | 0.0276 |
| JHU | SUPERIOR_FRONTAL_WM_L | freefluid | ISOVF | 0.0273 |
| JHU | GLOBUS_PALLIDUS_L | freefluid | ISOVF | 0.0253 |
| AAL | Vermis_4_5 | freefluid | V-CSF | 0.0243 |
| AAL | Cerebelum_4_5_R | freefluid | V-CSF | 0.0240 |
| AAL | Cerebelum_Crus1_L | freefluid | V-CSF | 0.0236 |
| JHU | GLOBUS_PALLIDUS_R | freefluid | ISOVF | 0.0232 |
| JHU | MIDDLE_FRONTO_ORBITAL__WM_L | freefluid | ISOVF | 0.0218 |
| JHU | Anterior_corona_radiata_R | freefluid | ISOVF | 0.0202 |
| JHU | INFERIOR_OCCIPITAL_WM_L | freefluid | ISOVF | 0.0200 |
| AAL | Cerebelum_Crus1_L | freefluid | ISOVF | 0.0198 |
| AAL | Hippocampus_R | freefluid | ISOVF | 0.0191 |
| JHU | Posterior_limb_of_internal_capsule_R | freefluid | ISOVF | 0.0189 |
| AAL | Cingulum_Ant_R | freefluid | V-CSF | 0.0179 |
| AAL | Parietal_Sup_R | freefluid | V-CSF | 0.0178 |
| AAL | Cerebelum_10_R | freefluid | ISOVF | 0.0175 |
| AAL | Cerebelum_Crus2_L | freefluid | V-CSF | 0.0171 |
| JHU | RECTUS__WM_L | freefluid | ISOVF | 0.0166 |
| AAL | Supp_Motor_Area_R | freefluid | V-CSF | 0.0154 |
| AAL | Cerebelum_6_L | freefluid | V-CSF | 0.0153 |
| JHU | Fornix__column_and_body__R | freefluid | ISOVF | -0.0158 |
| AAL | Temporal_Pole_Mid_L | freefluid | V-CSF | -0.0165 |
| JHU | Cingulum__cingulate_gyrus__R | freefluid | ISOVF | -0.0167 |
| JHU | PRE_CUNEUS_WM_R | freefluid | V-CSF | -0.0168 |
| AAL | Cerebelum_3_L | freefluid | ISOVF | -0.0168 |
| JHU | Fornix_cres__Stria_terminalis__R | freefluid | ISOVF | -0.0170 |
| AAL | Cerebelum_3_L | freefluid | V-CSF | -0.0175 |
| JHU | Tapatum_R | freefluid | V-CSF | -0.0177 |
| JHU | Pontine_crossing_tract__L | freefluid | V-CSF | -0.0181 |
| JHU | THALAMUS_R | freefluid | V-CSF | -0.0184 |
| JHU | Superior_corona_radiata_R | freefluid | V-CSF | -0.0187 |
| AAL | Temporal_Pole_Mid_R | freefluid | V-CSF | -0.0196 |
| JHU | Genu_of_corpus_callosum_L | freefluid | V-CSF | -0.0200 |
| AAL | Frontal_Inf_Orb_R | freefluid | V-CSF | -0.0200 |
| AAL | Frontal_Inf_Orb_L | freefluid | ISOVF | -0.0201 |
| AAL | Frontal_Inf_Oper_R | freefluid | V-CSF | -0.0218 |
| AAL | Occipital_Inf_L | freefluid | ISOVF | -0.0231 |
| AAL | Vermis_1_2 | freefluid | ISOVF | -0.0235 |
| AAL | Cerebelum_3_R | freefluid | V-CSF | -0.0246 |
| AAL | Vermis_1_2 | freefluid | V-CSF | -0.0246 |
| AAL | Frontal_Inf_Orb_L | freefluid | V-CSF | -0.0247 |
| AAL | Cerebelum_3_R | freefluid | ISOVF | -0.0253 |
| JHU | HIPPOCAMPUS | freefluid | ISOVF | -0.0263 |
| JHU | Genu_of_corpus_callosum_R | freefluid | V-CSF | -0.0263 |
| AAL | Vermis_3 | freefluid | V-CSF | -0.0272 |
| JHU | Fornix_cres__Stria_terminalis__R | freefluid | V-CSF | -0.0281 |
| AAL | Vermis_3 | freefluid | ISOVF | -0.0285 |
| JHU | Genu_of_corpus_callosum_R | freefluid | ISOVF | -0.0300 |
| JHU | Cingulum__cingulate_gyrus__L | freefluid | V-CSF | -0.0303 |
| AAL | Occipital_Inf_L | freefluid | V-CSF | -0.0314 |
| JHU | THALAMUS_L | freefluid | V-CSF | -0.0325 |
| JHU | HIPPOCAMPUS_R | freefluid | ISOVF | -0.0327 |
| JHU | HIPPOCAMPUS_R | freefluid | V-CSF | -0.0340 |
| JHU | Cingulum__hippocampus__R | freefluid | V-CSF | -0.0347 |
| JHU | Corticospinal_tract_R | freefluid | ISOVF | -0.0352 |
